# White matter hyperintensities and self-reported depression in a sample of patients with chronic headache

**DOI:** 10.1007/s10194-012-0493-y

**Published:** 2012-10-19

**Authors:** Gianluca Serafini, Maurizio Pompili, Marco Innamorati, Andrea Negro, Martina Fiorillo, Dorian A. Lamis, Denise Erbuto, Francesco Marsibilio, Andrea Romano, Mario Amore, Lidia D’Alonzo, Alessandro Bozzao, Paolo Girardi, Paolo Martelletti

**Affiliations:** 1 Department of Neurosciences, Mental Health and Sensory Organs, Suicide Prevention Center, Sant’Andrea Hospital, Sapienza University of Rome, 1035-1039, Via di Grottarossa, 00189 Rome, Italy; 2 Department of Clinical and Molecular Medicine, Sapienza University of Rome and Regional Referral Headache Centre, Sant’Andrea Hospital, Rome, Italy; 3 Department of Radiology, Stroke and Neurovascular Regulation Lab, Harvard Medical School, Massachusetts General Hospital, Boston, MA USA; 4 Department of Psychiatry and Behavioral Sciences, Emory University School of Medicine, Atlanta, GA USA; 5 Division of Neuroradiology, Department of Neuroscience, Sant’Andrea Hospital, Sapienza University of Rome, Rome, Italy; 6 Department of Neuroscience, Rehabilitation, Ophthalmology, Genetics, Maternal and Child Health, Section of Psychiatry, University of Genova, Genova, Italy

**Keywords:** PWMHs, DWMHs, Chronic headache, Self-reported depression, Age

## Abstract

White matter hyperintensities (WMH) have been associated with mood disorders in psychiatric patients. In the present study, we aimed to assess whether WMHs are associated with depressive symptoms and different sensitivity of the behavioral inhibition (BIS), and activation (BAS) systems in patients with chronic headache. Participants were 85 adult outpatients (16 men and 69 women) with a diagnosis of chronic headache. All of the patients underwent brain magnetic resonance imaging (MRI) and were administered the BIS/BAS scales and the Center for Epidemiologic Studies Depression Scale. Above 40 % of patients had periventricular WMHs (PWMHs) and almost 98 % had deep WMHs (DWMHs). Patients with PWMHs reported fewer depressive symptoms than patients without PWMHs. Patients with more severe DWMHs (compared with patients with mild or without DWMH lesions) were older and reported lower scores on the drive dimension of the BIS/BAS scales. In multivariate analyses, patients with PWMHs were 1.06 times more likely to report fewer depressive symptoms than patients without PWMHs. WMH lesions in patients with chronic headache were associated with less depression severity.

## Introduction

White matter hyperintensities (WMHs) appear as hyperintense signals on T2-weighted magnetic resonance images (MRI) and represent ependymal loss and differing degrees of myelination in the brain [1, 2]. WMHs, depending on the localization, are commonly classified as periventricular white matter hyperintensities (PWMHs) and deep white matter hyperintensities (DWMHs). WMHs have been reported to be commonly associated with older age and cardiovascular risk factors such as hypertension and diabetes [3–5]. Degenerative changes in brain white matter have been reported to be associated with mood disorders and suicidal behavior both in children and young adults [6–13]; however, they seem not to be specific to first episode psychotic disorders [14]. Taylor and colleagues hypothesized that patients with WMHs may be at a higher risk for suicide and developing mood disorders due to a possible disruption of neuroanatomic pathways [15]. Mood regulation depends on the complex extensive connections between the prefrontal cortex, amygdala–hippocampus complex, thalamus and basal ganglia [16].

Studies in both clinical and community-based settings have demonstrated the association between migraine and a number of specific psychiatric disorders, particularly major affective disorders [17–23]. Previous researchers have hypothesized that the relation between migraine and psychiatric disorder may be bi-directional given that migraine patients have a more than threefold risk of developing depression and the presence of depression is also associated with a greater risk of developing a migraine [21, 24].

Since the 1990s, brain MRI studies have investigated the presence of focal hyperintense abnormalities in patients with migraine [25]. Chronic migraine has been associated with the presence of white matter hyperintensities and migraine attack frequency, and attack history may be considered as a contributor of brain abnormalities in migraineurs [25]. In a sample of 28 patients with migraine, using high-resolution T1- and diffusion-weighted MRI and optimized voxel-based morphometry to localize gray and white matter density, Schmitz et al. [26] found that regions of brain abnormalities in migraineurs were mainly located in the frontal lobes, brainstem, and the cerebellum, and both attack frequency and disease duration predicted brain damage in migraine patients. To date, although the cause of migraine, particularly with aura, is commonly recognized as ischemic, which is consistent with its association with vascular risk factors [25], the natural history of white matter abnormalities in migraine patients is not completely understood. The relation between increased volume of white matter hyperintensities and a history of severe headache is also a matter of debate.

The structures and functions of a major motivational system, the Behavioral Inhibition System (BIS), have been described by Gray [27] in 1994. It has been suggested that the BIS modulates behavioral inhibition, anxiety, attention, and arousal towards signals of fear stimuli and punishment [28, 29]. A strong BIS is indicative of anxiety and inhibition even if few fear stimuli are present, whereas a weak BIS may not contribute to anxiety and inhibition even when severe stimuli occur. Gray [27, 28] also described the behavioral activation system (BAS) as a regulatory system measuring positive affect and approach behavior towards signals of reward. BAS activity may trigger positive affect and increase the likelihood of incentive acquisition. Refractoriness to incentives, low positive affect, and an absence of environmental engagement may be associated with a weak BAS [30].

As suggested by Gray [27, 28], the BIS may be considered an anxiety system and its activity may be investigated in several psychiatric conditions [31]. The BIS scale [32] includes seven items and assesses the tendency to respond with negative affect, anxiety, or fear in response to threatening events. The BAS scales [32] describe the tendency to respond with positive affect and motivation when faced with incentives or rewards. To our knowledge, no studies have investigated the sensitivity of the BIS and activation of the BAS concurrently with self-reported depression among patients with WMHs and chronic migraine.

In the present study, we aimed to assess whether WMHs are associated with self-reported depressive symptoms and different sensitivity of the BIS and BAS systems in patients with chronic headache.

## Methods

### Participants

Participants were 85 adult outpatients (16 men and 69 women) admitted at the Regional Referral Headache Centre, Department of Medical and Molecular Sciences, Sapienza University of Rome, Italy, between June 2010 and June 2011. Inclusion criteria were 18 years of age or older and a diagnosis of primary headache. Exclusion criteria included the presence of any psychiatric disorders or any condition affecting the ability of the patient to complete the assessment, including the denial of informed consent. The mean age of the sample was 50.13 ± 12.83 years (min/max 20/88 years). Table 1 summarizes the headache diagnoses as per the international classification of headache disorder (ICHD-II, ICHD-IIR).

**Table 1:** Diagnoses in the 85 study subjects (ICHD-II, ICHD-IIR)

	Frequency^a^	%
Chronic migraine	69	81.1
Migraine without aura	12	14
Migraine with aura	7	8.2
Episodic tension-type headache	4	4.7
Chronic tension-type headache	3	3.5

^a^Some patients presented multiple diagnoses

All the patients voluntarily participated in the study and provided written informed consent. The design of the study was approved by the Hospital’s Ethics Committee.

### Measures: clinical assessment

All the patients were administered the BIS/BAS scales and the Center for Epidemiologic Studies Depression Scale (CES-D).

### BIS/BAS scales

The BIS scale [32] includes seven items and measures the tendency to respond with negative affect, anxiety, or fear in response to threatening events. An example of an item on the BIS is “If I think something unpleasant is going to happen I usually get pretty ‘worked up’.” The BAS scales [32] describe the tendencies to respond with positive affect and motivation when faced with incentives or rewards. The BAS includes three distinct subscales: reward responsiveness, drive, and fun seeking. Individuals scoring high on the drive subscale are strongly motivated towards specific goals. The tendency to respond with elevated energy and positive affect when desired events are experienced was measured by the reward responsiveness scale. The fun-seeking subscale assessed the impulsive behavioral desire of fun conditions. Adequate psychometric properties (internal consistency, factor structure, test–retest reliability) of the BIS/BAS scales have been demonstrated [32–34]. Normative data were derived by a major community sample [34].

### CES-D scale

The CES-D scale is a short self-report scale designed to measure current level of depressive symptomatology in the general population, with an emphasis on the affective component. The 20 items were selected from previously validated depression scales. Participants were asked to rate each item on a scale from 0 (rarely or none of the time: less than 1 day) to 3 (most or all of the time 5–7 days) on the basis of how often they have felt that way during the previous week. The validity of the scale as a screening tool for depression has been documented in several populations [35–42]. In our sample, the Cronbach alpha for the CES-D was 0.86.

### Magnetic resonance image acquisition and rating of white matter hyperintensities

Brain MRIs were performed using a Siemens Sonata equipment, Erlangen, Germany (1.5 T). FLAIR scan sequence was used for WMH measurement (ax: TR 10000; TE 125; thickness 5 mm; matrix 144 × 256). Proton density and T2-weighted images were obtained (PD and T2 ax: TR 2870; TE 13/107; thickness 5 mm; matrix 147 × 256) in the axial and the coronal planes. Axial and sagittal T1-weighted images were also obtained (T1 ax: TR 647; TE 17; thickness 5 mm; matrix 128 × 192 T1 sag: TR 552; TE 17; thickness 5 mm; matrix 231 × 192). The presence of WMH was assessed by a neuroradiologist blind to all clinical information, using the modified Fazekas four-point rating scale which describes MRI hyperintensities on an ascending scale of intensity and frequency [43]. A second neuroradiologist, blind to all clinical information and previous ratings, reviewed all MRI films. The mean* k* value for interrater reliability for both PWMHs and DWMHs was 0.90. DWMHs and PWMHs as assessed by the Fazekas modified scale are summarized in Table 2.

**Table 2:** DWMHs and PWMHs as assessed by the Fazekas modified scale

	PWMHs	DWMHs
0	57.6	2.4
1	30.6	56.5
2	8.2	34.1
3	3.5	7.1

### Statistical analysis

A series of *t* tests and one-way Fisher exact tests were used to assess differences between groups. All the variables significant at the bivariate analyses were inserted as independent variables in a generalized linear model with a robust estimator. Groups were inserted as criterion. Associations between variables are reported as odds ratios (OR). All of the analyses were performed with the statistical package for social sciences SPSS 19.0 for Windows.

## Results

Above 40 % of patients had PWMHs and almost 98 % had DWMHs (see Table 2). Due to the low number of participants included in some categories, we collapsed levels of severity of white matter hyperintensity into two classes: (1) periventricular lesions were categorized in absent/present; and (2) deep lesions assessed as 0 and 1 at the Fazekas were collapsed together while levels 2 and 3 were combined.

Patients with PWMHs differed from those without periventricular lesions on depression severity (*t*_77.76_ = 2.30; *P* < 0.05) (Table 3). Patients with PWMHs had lower CES-D scores (13.79 ± 7.51 vs. 18.19 ± 9.68) than patients without PWMHs. Groups did not differ in age (*P* = 0.37), sex (*P* = 0.17), or for scores on the BIS/BAS scales.

**Table 3:** Differences between groups

	PWMHs				DWMHs			
	Absence of PWMHs	Presence of PWMHs	Test	*P*	No DWMHs or mild lesions	Moderate to severe DWMHs lesions	Test	*P*
Men	14.3 %	25.0 %		<0.17	20.0 %	17.1 %		<0.48
Age	49.02 ± 12.76	51.58 ± 12.96	*t*_83_ = −0.90	<0.37	47.40 ± 11.91	53.89 ± 13.26	*t*_83_ = −2.34	<0.05
CES-D	18.19 ± 9.68	13.79 ± 7.51	*t*_77.76_ = 2.30	<0.05	15.69 ± 8.35	17.47 ± 10.13	*t*_83_ = −0.86	<0.39
CES-D ≥ 16	52.1 %	36.4 %		<0.12	44.9 %	46.9 %		<0.52
Drive	10.49 ± 2.60	10.89 ± 2.89	*t*_83_ = −0.67	<0.51	11.14 ± 2.52	9.97 ± 2.86	*t*_83_ = 1.99	<0.05
Fun seeking	10.16 ± 2.50	10.19 ± 2.62	*t*_83_ = −0.06	<0.96	10.44 ± 2.45	9.80 ± 2.63	*t*_83_ = 1.15	<0.25
Reward responsiveness	16.76 ± 2.43	16.97 ± 2.25	*t*_83_ = −0.42	<0.68	17.24 ± 2.11	16.29 ± 2.57	*t*_83_ = 1.88	<0.06
BIS	22.16 ± 3.41	22.53 ± 3.20	*t*_83_ = −0.50	<0.62	22.34 ± 3.09	22.29 ± 3.64	*t*_83_ = 0.07	<0.94

Groups with different severity of DWMH lesions differed in age (*t*_83_ = −2.34; *P* < 0.05) and on the mean scores of the drive dimension of the BIS/BAS scales (*t*_83_ = 1.99; *P* < 0.05). Specifically, patients with more severe DWMHs were older (53.89 ± 13.26 vs. 47.40 ± 11.91) and reported lower scores on the drive dimension (9.97 ± 2.86 vs. 11.14 ± 2.52) than patients with mild or without any deep lesion.

The variables found to be significant at the bivariate level were inserted as independent variables in two generalized linear models with groups as dependent variables (see Table 4, 5). The models fit the data well (PWMH: Likelihood ratio *χ*_1_^2^ = 5.83; *P* < 0.05; DWMH: Likelihood ratio *χ*_2_^2^ = 8.24; *P* < 0.05). Patients with PWMHs were 1.06 times more likely to have lower CES-D scores (*P* < 0.05) than patients without PWMHs. Patients with more severe DWMHs were 1.04 times more likely to be older (*P* < 0.05) than patients with mild or without any DWMH lesions.

**Table 4:** Generalized linear model: criterion PWMHs

Parameter	*B*	Std. error	95 % Wald confidence interval		Hypothesis test			95 % CI		
			Lower	Upper	Wald Chi-square	*df*	Sig.	OR	Lower	Upper
CES-D	−0.07	0.03	−0.13	−0.01	4.66	1	0.05	0.94	0.88	0.99

Model fit: Likelihood ratio *χ*_1_^2^ = 5.83; *P* < 0.05; AIC = 63.65; AICC = 63.81; Pearson *χ*_31_^2^ = 32.58; Value/g dl = 1.05

**Table 5:** Generalized linear model: criterion DWMHs

Parameter	*B*	Std. error	95 % Wald confidence interval		Hypothesis test			95 % CI		
			Lower	Upper	Wald Chi-square	*df*	Sig.	OR	Lower	Upper
Drive	−0.16	0.09	−0.33	0.02	3.15	1	0.08	0.86	0.72	1.02
Age	0.04	0.02	0.01	0.08	5.23	1	0.05	1.04	1.01	1.08

Model fit: Likelihood ratio *χ*_2_^2^ = 8.24; *P* = 0.05; AIC = 109.02; AICC = 109.33; Pearson *χ*_77_^2^ = 81.15; Value/g dl = 1.05

## Discussion

Our aim was to assess whether WMHs were associated with depressive symptoms and sensitivity of the BIS and BAS systems in patients with chronic headache. Our findings suggest that differences in brain lesions are associated with differences in age and self-reported depression severity as measured by the CES-D.

Specifically, in multivariate analyses, individuals with PWMHs were more likely to report fewer depressive symptoms as compared with patients without PWMHs. These findings contradict our previous results regarding the association between white matter abnormalities and both unipolar and bipolar depression [8, 11, 14]. However, in our previous samples, we investigated patients with specific psychiatric conditions such as unipolar and bipolar disorders, whereas the current sample includes only individuals with chronic migraine without any evidence of comorbid psychiatric disorders. This may suggest that the association between WMHs and higher levels of depression is specific to particular subgroups of patients who have a psychiatric disorder [11, 12, 14], and it may not be a factor among inpatients with medical conditions.

Whether the presence of WMHs predicts a poor prognosis in specific at-risk subgroups of patients has not been clearly established. Taylor et al. [44] found a relation between the progression and severity of WMHs and negative long-term outcomes in depressed patients. Prefrontal WMHs have been associated with cognitive dysfunctions in depressed older patients, poor or delayed antidepressant response [45], and poor treatment outcomes were associated with subcortical and basal ganglia, but not periventricular hyperintensities [46]. In contrast, there have been studies which reported no differences in treatment response and functional outcomes between elderly patients with and without WMHs [47, 48].

In our sample, those patients with more severe DWMHs differed from patients with mild or no DWMH lesions, as they were more likely to be older. Previous studies have strongly suggested that patients with migraine, particularly those diagnosed as having migraine with aura and at least 12 migraine episodes per year [49–51], are at an increased risk (with an odds ratio of 3.9) for subclinical brain infarct-like lesions, even after controlling for the most common cardiovascular risk factors and a history of cardiovascular diseases [52]. Unfortunately, we only examined the effect of age and did not consider whether patients with more pronounced cardiovascular risk factors were more likely to have DWMHs.

Although DWMHs have been suggested to have mainly a vascular origin, recent studies have reported that these brain lesions may reverse over time raising questions about their hypothetic ischemic origin [53, 54]. It has been demonstrated that frequent migraine attacks may be associated with increased iron concentration/accumulation in deep nuclei which is involved in central pain processing. These findings suggest that iron accumulation might have a possible role in determining chronic migraine [49]. Other hypotheses have been proposed to explain the existence of white matter abnormalities in migraineurs although definitive conclusions are still not established. It has been suggested that recurrent migraine attacks may affect vulnerable deep tissues penetrating arteries and contributing to local critical hypoperfusion leading to minor brain injury observed as white matter abnormalities. Other proposed mechanisms have been suggested including atherosclerotic insults associated with the presence of cardiovascular risk factors [55, 56], endothelial dysfunction (e.g., both endothelial activation and impaired vascular reactivity) [57–59], genetic risk factors which are common in migraine and stroke [60], drugs commonly used to treat headache, having vasoconstrictor activity [61], and cardiac dysfunctions such as patent foramen ovale. In addition, irrespective of whether endothelial dysfunction is linked to platelet aggregation, recurrent endothelial insults may lead to microvascular brain damage contributing to white matter lesions [62].

The present study must be considered in the light of the following limitations. The small sample size limits the generalizability of the current findings. In addition, our MRI study was limited by low spatial resolution and performed using only a 1.5 T MRI scanner. Studies using a 3 T MRI scanner having a higher resolution are able to detect a higher number and more precise extents of WMHs. An analysis quantifying total white matter lesion volume would strengthen the findings. Diffusion tensor imaging techniques may be more sensitive in detecting white matter abnormalities in association with mood disorders. Recently, some authors [63] have demonstrated ‘‘occult’’ brain damage in migraine patients using diffusion-weighted MRI, particularly tractography.

Furthermore, the Fazekas modified rating scale, which was used to measure the frequency and intensity of white matter lesions, is a quantitative lesion assessment method that may be limited since it is a visual rating scale less objective than many volumetric methods available. Also, we have not systematically assessed clinical disease course, age, gender, comorbidities, or history of substance abuse and dependence in the individuals recruited. In addition, some limitations regarding the utilization of the CES-D must be mentioned. Lack of consistency in some item responses in the measurement of depression suggests that response patterns may be affected by unique conceptualizations of depression among different ethnic groups [64]. Although the stability and applicability of the CES-D Scale with various ethnic groups have been widely assessed, few studies have addressed the cross-contextual generalizability of the CES-D Scale.

## Conclusion

Overall, patients with chronic headache having PWMHs were more likely to have lower self-reported depression than those without PWMHs, and patients with more severe DWMHs were more likely to be older than those with less severe DWMHs. Further studies are needed to investigate the relation between progression of migraine and onset of new white matter lesions while considering the possible association between migraine attack rate and progression of lesion impairment [25], as well as the link between white matter lesions, major affective disorders, and migraine attacks over time. Also, functional impairment related to the presence of self-reported depression in patients with chronic migraine should be considered in terms of long-term cognitive changes. Importantly, different mechanisms may be considered in the emergence of WMHs and it is possible that WMHs may represent only the ‘tip of the iceberg’ in terms of structural white matter lesions.
